# Serum Homocysteine Level Is a Predictor of Relapse and Prognosis in Patients With First-Attack Neuromyelitis Optica Spectrum Disorders

**DOI:** 10.3389/fneur.2021.667651

**Published:** 2021-05-26

**Authors:** Jinwei Zhang, Yanfei Li, Yongyan Zhou, Yi Zhao, Haojie Xie, Ranran Duan, Yaobing Yao, Zhe Gong, Junfang Teng, Yanjie Jia

**Affiliations:** Department of Neurology, The First Affiliated Hospital of Zhengzhou University, Zhengzhou, China

**Keywords:** expanded disability status scale, neuromyelitis optica spectrum disorders, prognosis, relapse, serum homocysteine

## Abstract

**Background:** Many patients with neuromyelitis optica spectrum disorders (NMOSD) experience the adverse consequences of relapse and disability aggravation. Thus, it is necessary to identify sensitive and reliable biomarkers for early prognosis. This study investigated whether serum homocysteine (Hcy) level was associated with the risk of relapse or poor prognosis in first-attack NMOSD patients.

**Methods:** We enrolled 161 first-attack NMOSD patients in this retrospective study. We reviewed their medical records and evaluated their initial Expanded Disability Status Scale (EDSS). Clinical outcomes were measured by the final EDSS and the relapse rate. The association between Hcy levels and EDSS score at last follow-up was analyzed by binary logistic regression. The association between Hcy levels and relapse rate was assessed by Cox regression analysis. Receiver operating characteristic (ROC) curve analysis was used to predict the target value of Hcy reduction.

**Results:** Compared with the high Hcy group, the final EDSS score in the low Hcy group was significantly lower (median: 0.5 vs. 2.5, *P* < 0.001). The relapse rate differed significantly between these groups (30.6 vs. 50.0%, *P* = 0.023). Multivariate analysis showed that the initial EDSS score (odds ratio [OR] 3.03, 95% confidence interval [CI] 2.07–4.45, *P* < 0.001) and serum Hcy level (OR 1.13, 95%CI 1.04–1.22, *P* = 0.002) were significantly associated with poor prognosis in NMOSD patients. Additionally, multivariate analysis showed that serum Hcy level (hazard ratio 1.06, 95%CI 1.04–1.09, *P* < 0.001) was an independent predictor of the risk for relapse in NMOSD. The 12-month relapse rate of the high Hcy group was 34.8%, and 50% of high Hcy patients relapsed within 35 months after the first onset. A serum Hcy level exceeding 14.525 μmol/L indicated a high risk of relapse, with a sensitivity of 43.7%, specificity of 90.0%, and area under the ROC curve of 0.674 (95%CI 0.59–0.76, *P* < 0.001).

**Conclusion:** Serum Hcy level is an independent predictor of relapse and poor prognosis in first-attack NMOSD patients. Early monitoring and reduction of serum Hcy levels may be of great significance in the prevention of disease relapse and severe disability.

## Introduction

Neuromyelitis optica spectrum disorder (NMOSD) is a rare and severe, inflammatory, immune-mediated, demyelinating disease of the central nervous system (CNS). It is commonly characterized by monophasic or recurrent optic neuritis, longitudinal extensive transverse myelitis, or posterior region syndrome (1, 2). The pathogenesis of diseases is closely related to a circulating antibody against the astrocytic aquaporin 4 (AQP4) water channel protein (3, 4). At present, there is no validated consensus on NMOSD treatment, although empirical use of corticosteroids, immunosuppressants, and plasma exchange are considered effective treatments for both the first-attack and relapses of the disease (5, 6). However, many patients still face the adverse consequences of disease relapse and increased disability (7–9). Therefore, the determination of sensitive and reliable biomarkers for predicting the prognosis of the disease and implementing early treatment measures to reduce the risk of relapse is of great significance (10).

Homocysteine (Hcy) is a non-essential sulfur-containing amino acid and is an important intermediate product of folic acid, vitamin B12, and single carbon metabolism (11). Hcy level is considered as an independent risk factor for many cardiovascular and cerebrovascular diseases (12). The nervous system is particularly sensitive to extracellular Hcy, which induces oxidative stress by increasing the production of reactive oxygen species (ROS), and promotes excitotoxicity by stimulating the N-methyl-D-aspartate (NMDA) receptor, causing neuronal damage (13, 14). In addition, Hcy is seen to be able to induce the destruction of the blood–brain barrier (BBB) and to regulate the function of adaptive immune system cells (15–17). Recently, the role of Hcy in many autoimmune diseases has attracted great attention of researchers, particularly demyelinating diseases of the nervous system, such as multiple sclerosis (18). Hcy levels in patients with multiple sclerosis was higher than that of healthy people, and hyperhomocysteinemia may be related to disease progression and disability severity in patients with multiple sclerosis (19, 20).

A previous study has shown that serum Hcy levels were increased significantly in patients with NMOSD with an Expanded Disability Status Scale (EDSS) score ≥ 4 in the acute stage, which indicates that Hcy may play a role in the pathogenesis or progression of NMOSD (21). However, it is not clear whether Hcy levels are related to relapse and poor prognosis in this disease. In our study, we investigated the association of Hcy levels with relapse risk and prognosis of first-attack NMOSD, and sought to determine the target value of Hcy level for predicting risk of relapse in NMOSD patients.

## Materials and Methods

### Patients

**Figure 1:**
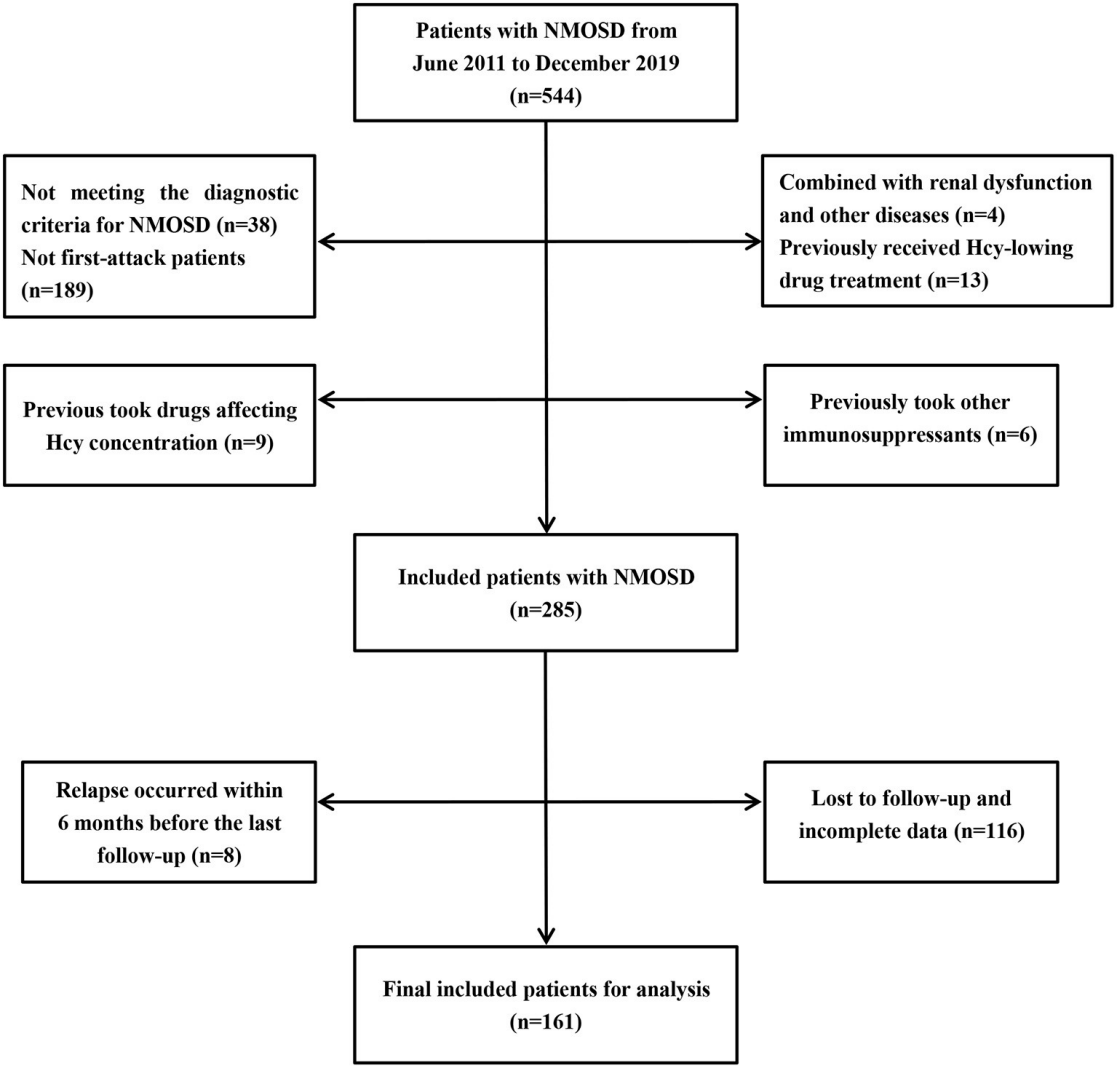
Patients selection process. NMOSD, neuromyelitis optica spectrum disorders; Hcy, homocysteine.

In this retrospective study, we collected 544 cases of NMOSD treated in the First Affiliated Hospital of Zhengzhou University from June 2011 to December 2019. The inclusion criteria were as follows: (1) meeting the 2006 or 2015 internationally recognized diagnostic criteria for NMOSD (22, 23); (2) confirmed as first-attack NMOSD patients; (3) no diseases involving renal dysfunction, vitamin B12 dysfunction, hypothyroidism, or hemolysis; (4) no homocysteine-lowering drug treatment before admission; (5) no use of drugs affecting Hcy concentration, such as phenytoin, isoniazid, medroxyprogesterone, and levodopa, before admission (24); (6) no immunosuppressive therapy in the 6 months before admission, although use of immunomodulators (such as interferon-beta) was not excluded; (7) no relapse within 6 months before the last follow-up; (8) availability of complete data. In total, 161 NMOSD patients met these criteria and were included in the study. The detailed selection process is shown in Figure 1.

This study was approved by the Ethics Committee of Zhengzhou University (2019-KY-018). Written informed consent was provided by all patients or their proxy.

### Data Collection

We reviewed medical records and collected individual clinical data, including gender, onset age, medical history, anti-AQP4 status, treatment plan, and serum Hcy, folic acid, and vitamin B12 levels. The EDSS was preformed and recorded as the initial EDSS scores.

Blood samples were collected from peripheral veins of all NMOSD patients after overnight fasting at 7:00–8:00 am the next day and were kept on ice prior to separation (maximum 30 min after drawing). Hcy, folic acid, and vitamin B12 levels in these samples were determined in the coagulation function laboratory of the First Affiliated Hospital of Zhengzhou University. Hcy levels were assessed by rate method with an automated chemistry analyzer (Roche Group, Sweden). Hyperhomocysteinemia was defined as homocysteine concentration ≥10 μmol/L (25, 26). Serum folic acid and vitamin B12 levels were measured with a chemiluminescent immunoassay on the Maglumi 2000 Plus analyzer (Shenzhen New Industries Biomedical Engineering Company Limited, China). Serum or cerebrospinal fluid samples were used to detect anti-AQP4 status at the Neurology Laboratory of the First Affiliated Hospital of Zhengzhou University using an assay of live cells transfected with AQP4. All tests were performed according to the manufacturers' instructions, and the investigators were blinded to the patients' diagnoses and clinical symptoms.

### Clinical Outcomes

The primary outcome of this study was the relapse rate. Relapse referred to the occurrence of new, progressive, or recurrent neurological symptoms in patients with NMOSD, which lasted for at least 24 h, and which increased the existing EDSS score by 0.5 points (27). The relapse rate and first relapse duration were obtained by clinical or telephonic follow-up every 6 months.

The secondary outcome was clinical disability, determined according to the final EDSS score, which was determined by an experienced neurologist at the last follow-up. To reduce the error of transition of EDSS score from 0 to 1.0, patients with an initial EDSS score of 0 needed an increase of 1.5 points, patients with score of 1.0–5.0 needed an increase of 1.0 points, and patients with a score > 5.0 needed an increase of 0.5 points. The final EDSS were converted into categorical variables. Patients with EDSS score > 3 were defined as showing poor recovery, and those with EDSS scores ≤ 3 were defined as showing good recovery.

### Statistical Analysis

The data were analyzed using SPSS 26.0 software (International Business Machines Corporation, Chicago, IL, USA). The graphs were generated by using GraphPad Prism 8.3 (Graphpad Inc, Harvey Motulsky, USA). Although Hcy levels could be regarded as a continuous variable, they were stratified into high Hcy levels (≥10 μmol/L) and normal Hcy levels (<10 μmol/L) (25, 26). We compared the demographic and clinical characteristics between patients with high or normal Hcy levels. If the Kolmogorov–Smirnov test showed that continuous variables were normally distributed; they were expressed as means and standard deviations (SD) and were compared by *t*-tests. Otherwise, data were expressed as medians (interquartile range) and were compared using a Mann–Whitney *U*-test. Classification variables were expressed as frequency (percentage, %) and were compared by a chi-square test or Fisher's exact test. Spearman's correlation analysis was used to evaluate the relationship between Hcy level and EDSS progression. A univariate logistic regression analysis was used to evaluate the factors potentially related to poor recovery in first-attack NMOSD patients. The variables with *P* < 0.2, which were clinically considered to be closely related to the dependent variables, were included in a multivariate model. A multivariate logistic regression was preformed to analyze the independent effect of Hcy level on recovery. The results were expressed using odds ratios (ORs). A Kaplan–Meier analysis and log-rank test were used to analyze the effect of Hcy levels in different groups on the timing of first relapse. A univariate Cox proportional hazards model was used to screen variables with *P* ≤ 0.2, and a multivariate Cox regression model was used to analyze the predictive strength of Hcy levels on the timing of the first relapse of NMOSD. The results were expressed by hazard ratios (HRs). Receiver operating characteristic (ROC) curve analysis was used to test the predictive ability of Hcy for disease relapse and to predict the optimal cutoff value for reduction of Hcy level in patients with first-attack NMOSD. A two-tailed *P* < 0.05 was considered statistically significant.

## Results

### Clinical and Demographic Characteristics

In this cohort study, 161 NMOSD patients were included. The detailed inclusion and exclusion criteria are shown in Figure 1. The average onset age was 45.17 ± 15.17 years. More than three-quarter patients were women. The median follow-up time was 42.2 (27.2–55.9) months. Anti-AQP4-positive cerebrospinal fluid or serum was identified in almost half of the patients.

Patients were divided into a normal Hcy group (serum Hcy level < 10 μmol/L; *n* = 49) and a high Hcy group (serum Hcy level ≥ 10 μmol/L; *n* = 112). Compared with the high Hcy group, the proportion of female patients and the level of folic acid in the normal Hcy group were significantly higher (*P* = 0.002). Other parameters, including onset age and vitamin B12 level, showed no significant difference between these groups. Additionally, the number of patients with hypertension, diabetes, or autoimmune diseases were not statistically significantly different between the groups. Patients received different treatments according to clinical symptoms and economic status, such as corticosteroids, immunosuppressants (mycophenolate mofetil, azathioprine, methotrexate, tacrolimus, and rituximab), and received rehabilitation training, but there was no statistically significant differences in these parameters between the two groups. There was also no significant difference in the initial EDSS score between the two groups. At the last follow-up, the final EDSS score of the normal Hcy group was significantly lower than that of high Hcy group (*P* < 0.001), while the proportion of good recovery was higher in the former than in the latter group (*P* = 0.018). The relapse rate differed significantly between the two groups (*P* = 0.023). The demographic and clinical features of the patients are summarized in Table 1.

**Table 1:** Demographic and clinical characteristics associated with categorized Hcy.

**Clinical characteristics**	**Total patients (*n* = 161)**	**Normal Hcy group (*n* = 49)**	**High Hcy group (*n* = 112)**	***P*-value**
**Demographic characteristics**				
Age of onset, years, mean ± SD	45.17 ± 15.17	45.53 ± 10.96	45.01 ± 16.73	0.815
Gender, female, *n* (%)	122 (75.8)	46 (93.9)	76 (67.9)	<0.001^*^
**Medical history**				
Hypertension, *n* (%)	22 (13.7)	5 (10.2)	17 (15.2)	0.398
Diabetes, *n* (%)	12 (7.5)	1 (2.0)	11 (9.8)	0.160
Autoimmune diseases, *n* (%)	21 (13.0)	10 (20.4)	11 (9.8)	0.066
**Anti-AQP4 status**, ***n*** **(%)**				
Positive	78 (48.4)	22 (44.9)	56 (50.0)	0.551
Negative	53 (32.9)	18 (36.7)	35 (31.3)	0.496
Not tested	30 (18.6)	9 (18.4)	21 (18.8)	0.954
**Disease characteristics**				
Initial EDSS,median (IQR)	5.0 (3.0–6.0)	5.0 (3.75–7.0)	5.0 (3.0–6.0)	0.309
Final EDSS, median (IQR)	1.5 (0.25–3.5)	0.5 (0–2.25)	2.5 (1.0–4.0)	<0.001^*^
Outcomes, good recovery, *n* (%)	114 (70.8)	41 (83.7)	73 (65.2)	0.018^*^
Follow-up time, month, median (IQR)	42.2 (27.2–55.9)	39.8 (23.1–55.9)	42.2 (29.8–55.3)	0.302
Relapse, *n* (%)	71 (44.1)	15 (30.6)	56 (50.0)	0.023^*^
**Therapy regimens**				
Corticosteroid treatment, *n* (%)	151 (93.8)	44 (89.8)	107 (95.5)	0.165
Immunosuppressant treatment, *n* (%)	59 (36.6)	18 (36.7)	41 (36.6)	0.988
Rehabilitation training, *n* (%)	8 (5.0)	4 (8.2)	4 (3.6)	0.483
Hcy,median (IQR)	11.1 (9.52–14.53)	8.32 (7.16–9.36)	13.2 (10.91–16.98)	<0.001^*^
Folic acid, median (IQR)	7.64 (4.94–12.94)	9.74 (6.85–13.94)	6.98 (4.43–11.83)	0.002^*^
Vitamin B12, median (IQR)	872.00 (464.00–2000.00)	837.00 (476.85–1995.00)	907.95 (455.75–2000.00)	0.561

*Continuous variables were presented as mean ± SD or median (IQR = 25th−75th percentile), and categorical variables were described as percentages. Normal Hcy Group, patients whose homocysteine levels were lower than 10 μmol/L; High Hcy Group, patients whose homocysteine levels were higher than or equal to 10 μmol/L. AQP4, aquaporin-4; EDSS, expanded disability status scale; Hcy, homocysteine*.

* *P < 0.05*.

### Predictors Associated With Good Outcomes for Patients With First-Attack NMOSD

**Figure 2:**
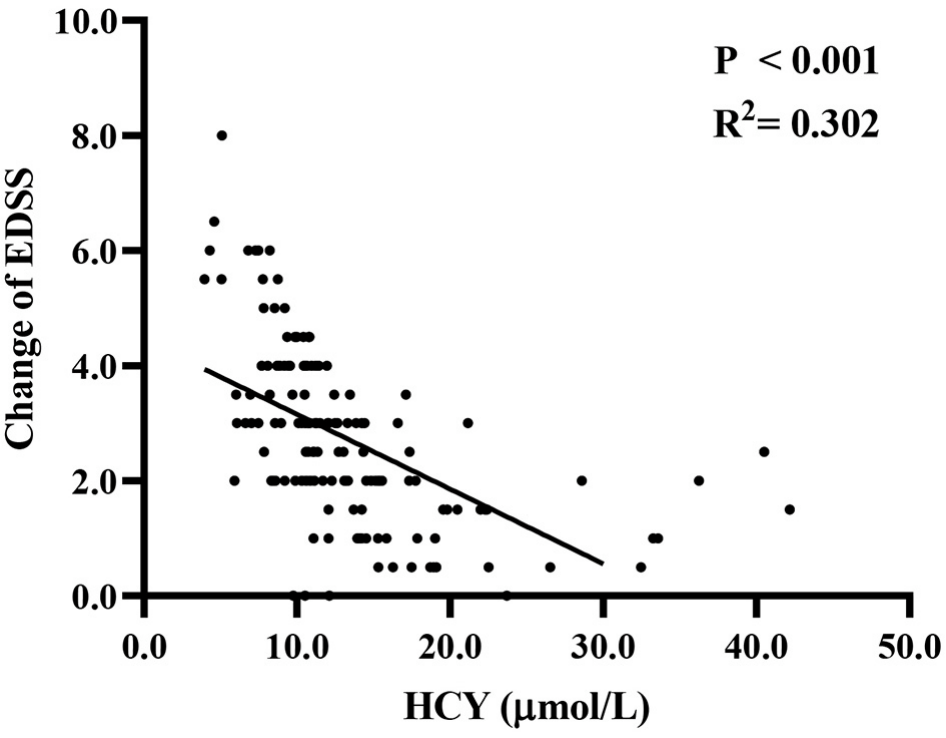
The association of Hcy level with changes between final EDSS and initial EDSS. EDSS, expanded disability status scale; Hcy, homocysteine.

Spearman's correlation analysis (Figure 2) was used to investigate the relationship between Hcy level and clinical recovery (assessed as final EDSS minus initial EDSS in the same patient). We found that the change in EDSS was negatively correlated with Hcy level (*r*^2^ = 0.302, *P* < 0.001), and the lower the level of Hcy, the greater the change in EDSS.

Univariate analysis (Table 2) showed that serum Hcy level was positively correlated with the final EDSS score (OR 1.07, 95%CI 1.01–1.13, *P* = 0.018). Initial EDSS score (OR 2.77, 95%CI 1.96–3.90, *P* < 0.001) and hypertension (OR 2.86, 95%CI 1.14–7.16, *P* = 0.025) were significantly correlated with prognosis. However, there was no correlation of age, gender, anti-AQP4 antibody status, other complications, folic acid or vitamin B12 levels, and treatment methods with the risk of poor prognosis in NMOSD patients. After selecting variables, and adjusting for age, hypertension, and initial EDSS score, multivariate logistic regression analysis showed that the initial EDSS score (OR 3.03, 95%CI 2.07–4.45, *P* < 0.001) and serum Hcy level (OR 1.13, 95%CI 1.04–1.22, *P* = 0.002) remained significantly correlated with poor prognosis in patients with NMOSD. The risk of poor recovery increased 1.13 times with every 1 μmol/L increase in Hcy level.

**Table 2:** Predictors of outcomes for NMOSD patients by univariate and multivariable logistic regression models.

**Factor**	**Univariate analysis**		**Multivariable analysis**	
	**OR (95% CI)**	***P*-values**	**OR (95% CI)**	***P*-values**
Age of onset	1.02 (1.00–1.05)	0.058	1.02 (0.99–1.05)	0.23
Gender, male	1.29 (0.59–2.81)	0.514		
Hypertension	2.86 (1.14–7.16)	0.025^*^	1.35 (0.36–5.13)	0.658
Diabetes	1.23 (0.35–4.31)	0.743		
Autoimmune diseases	0.53 (0.17–1.67)	0.279		
Anti-AQP4 status^a^	1.81 (0.82–4.00)	0.144		
Initial EDSS	2.77 (1.96–3.90)	<0.001^*^	3.03 (2.07–4.45)	<0.001^*^
Corticosteroid treatment	1.70 (0.35–8.31)	0.513		
Immunosuppressant	0.97 (0.48–1.97)	0.936		
treatment				
Rehabilitation training	0.80 (0.16–4.11)	0.789		
Hcy	1.07 (1.01–1.13)	0.018^*^	1.13 (1.04–1.22)	0.002^*^
Folic acid	1.00 (0.95–1.05)	0.962		
Vitamin B12	1.00 (0.99–1.00)	0.425		

*NMOSD, neuromyelitis optica spectrum disorders; OR, odds ratio; CI, confidence interval; AQP4, aquaporin-4; EDSS, expanded disability status scale; Hcy, homocysteine*.

a *Not-tested patients (n = 30) were excluded*.

* *P < 0.05*.

### Predictors Associated With Relapse in Patients With First-Attack NMOSD

**Figure 3:**
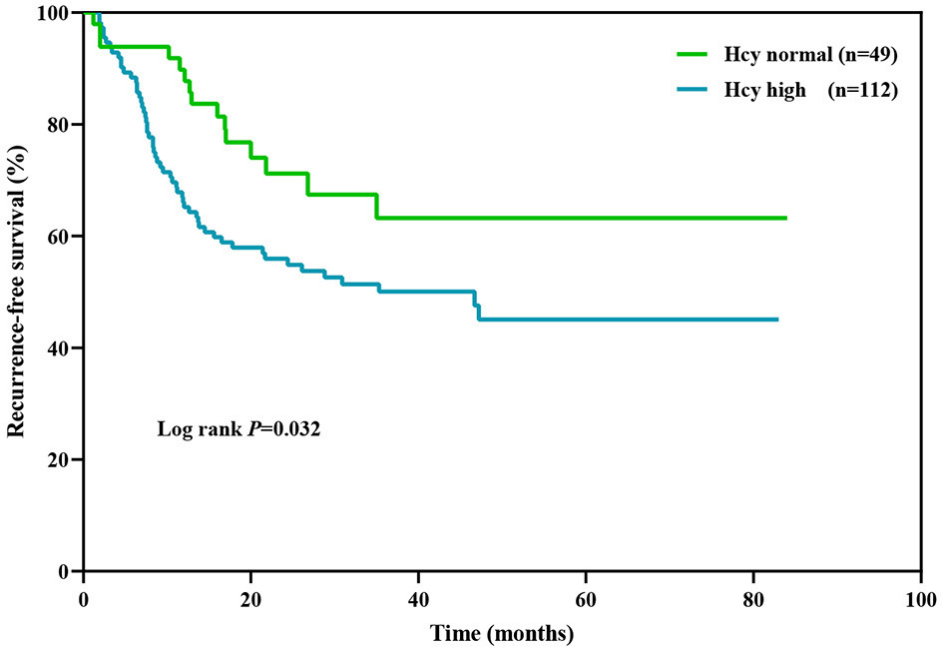
Kaplan–Meier analysis demonstrating the cumulative proportion of patients without relapse. Hcy, homocysteine.

Kaplan–Meier survival analysis (Figure 3) showed that Hcy level was a significant predictor of relapse rate in NMOSD patients (log rank test *P* = 0.035). The median relapse interval in the high Hcy group was 46.7 months, and the relapse rate within 12 months was 34.8%. Half of the high Hcy group patients relapsed within 35 months of the first onset.

The Cox proportional hazards model was used to test the independent effect of Hcy level on the relapse rate in NMOSD patients (Table 3). Univariate analysis showed that Hcy level (HR 1.07, 95%CI 1.04–1.09, *P* < 0.001) was significantly correlated with the risk of relapse. Hypertension (HR 1.58, 95%CI 0.86–2.89, *P* = 0.138), concomitant autoimmune disease (HR 0.43, 95%CI 0.17–1.07, *P* = 0.068), initial EDSS score (HR 1.11, 95%CI 0.98–1.27, *P* = 0.098), and folic acid level (HR 0.97, 95%CI 0.93–1.01, *P* = 0.193) had moderate effects, while age, gender, diabetes, anti-AQP4 antibody status, treatment methods, and serum vitamin B12 level had no correlation with relapse. After selecting variables, a multivariate Cox proportional hazards model showed that Hcy level (HR 1.06, 95%CI 1.04–1.09, *P* < 0.001) remained a risk factor for relapse in NMOSD patients.

**Table 3:** Predictors of relapse in NMOSD patients by univariate and multivariable Cox proportional hazards models.

**Factor**	**Univariate analysis**		**Multivariable analysis**	
	**HR (95% CI)**	***P*-values**	**HR (95% CI)**	***P*-values**
Age of onset	1.00 (0.98–1.01)	0.871		
Gender, male	1.03 (0.59–1.77)	0.935		
Hypertension	1.58 (0.86–2.89)	0.138	1.38 (0.74–2.58)	0.309
Diabetes	1.00 (0.40–2.48)	1.00		
Autoimmune diseases	0.43 (0.17–1.07)	0.068	0.57 (0.23–1.43)	0.227
Anti-AQP4 status^a^	0.91 (0.54–1.53)	0.713		
Initial EDSS	1.11 (0.98–1.27)	0.098	1.13 (0.99–1.29)	0.072
Corticosteroid treatment	1.30 (0.47–3.56)	0.613		
Immunosuppressant treatment	0.92 (0.56–1.49)	0.723		
Rehabilitation training	0.46 (0.11–1.86)	0.275		
Hcy	1.07 (1.04–1.09)	<0.001^*^	1.06 (1.04–1.09)	<0.001^*^
Folic acid	0.97 (0.93–1.01)	0.193	0.98 (0.95–1.02)	0.410
Vitamin B12	1.00 (1.00–1.00)	0.629		

*NMOSD, neuromyelitis optica spectrum disorders; HR, hazard ratio; CI, confidence interval; AQP4, aquaporin-4; EDSS, expanded disability status scale scores; Hcy, homocysteine*.

a *Not-tested patients (n = 30) were excluded*.

* *P < 0.05*.

**Figure 4:**
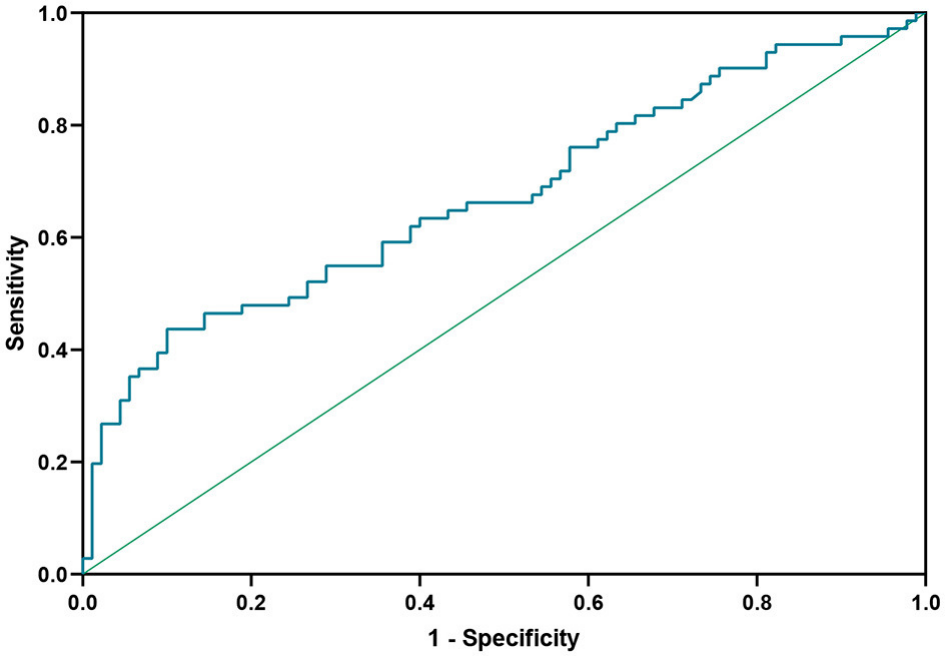
Receiver operating characteristic (ROC) curve showing the predictive ability of Hcy for disease relapse. Hcy, homocysteine.

ROC curve analysis was used to determine the target value for reduction of Hcy in NMOSD patients (Figure 4). At an Hcy cut-off value of 14.525 μmol/L, the sensitivity for predicting relapse was 43.7%, the specificity was 90.0%, and the area under ROC curve (AUC) was 0.674 (95%CI 0.59–0.76, *P* < 0.001).

## Discussion

In this cohort study, we investigated the possible association between Hcy level and disease prognosis through a large NMOSD data set. We found that compared with those with normal Hcy levels, patients with high Hcy levels had a higher relapse rate and worse recovery. The lower the Hcy level, the greater the change between the final and initial EDSS scores. Hcy level may thus be an independent risk factor for further relapse and poor prognosis. The optimal cut-off value of Hcy level for predicting relapse was 14.525 μmol/L.

There is increasing awareness of the role of serum Hcy in autoimmune demyelinating diseases of the CNS (13). Recent studies have shown that the serum Hcy levels of multiple sclerosis patients were higher than that of a healthy control group, by evaluating baseline levels and follow-up EDSS (19, 20). Compared with multiple sclerosis patients with a lower Hcy level, those patients with hyperhomocysteinemia showed more disease progression (28, 29). However, no studies to date had evaluated any association between serum Hcy and the progression and relapse of NMOSD. In our study, we found that patients with high serum Hcy showed a higher relapse rate and worse prognosis, and the relapse rate within 12 months was 34.8%. The changes of final EDSS scores from the initial EDSS scores were negatively correlated with Hcy levels, and the lower the level of Hcy, the greater the change in EDSS. Recently, it was reported that, in patients with acute NMOSD, the serum Hcy level of patients with severe initial symptoms (EDSS score ≥ 4) was significantly increased as compared with patients with mild initial symptoms (EDSS score < 4) (21). This finding, together with our data, supported the potential role of serum Hcy levels in the pathogenesis and disease progression of NMOSD and confirmed serum Hcy level as an independent risk factor of relapse and poor prognosis in patients with first-attack NMOSD.

The nervous system is particularly sensitive to extracellular Hcy, which may influence the prognosis of diseases through various mechanisms (30). Hcy in blood could induce oxidative stress and mitochondrial dysfunction by increasing ROS production (14, 31), and could stimulate NMDAR to promote excitotoxicity, which would lead to an increase in intracellular calcium levels and the activation of caspases, damaging neuronal DNA, and inducing apoptosis (19, 32, 33). In addition, Hcy is an important intermediate in the methionine cycle (13). High Hcy levels could reduce the availability of S-adenosylmethionine, which is an important methyl donor for many biochemical processes (29, 34). In the CNS, myelin basic protein (MBP) is the main component of myelin, and its methylation is very important for the maintenance and repair of the myelin sheath (35, 36). Hyperhomocysteinemia may lead to hypomethylation of MBP, which reduces its hydrophobicity and stability, thus leading to structural instability and degeneration and loss of the myelin sheath, with an adverse impact on disease progression (37, 38).

The pro-inflammatory effect of Hcy could also be mediated by regulation of cellular function in the adaptive immune system and destruction of the BBB. This could interfere with the reaction of T lymphocytes and B lymphocytes, natural killer cells, and adhesion molecules (15, 39, 40). Th17 cells interfere with the BBB by producing pro-inflammatory IL-17 and migrate to the CNS by expression of the chemokine receptor CCR-6 (CD196) (18). Hcy could indirectly influence the function of the BBB by promoting the differentiation of Th17 cells (41). The central event in NMOSD is the entry of anti-AQP4 antibody into the CNS through the damaged BBB, and its binding to AQP4 on astrocytes, which leads to inflammatory lesions and CNS injuries (42). There is evidence that serum Hcy itself could induce BBB destruction, thereby aggravating disease progression (17). At the same time, Hcy is also produced and secreted by astrocytes, and astrocyte activation increases secretion of Hcy, which may be one of the reasons for its abnormal levels in NMOSD patients (30).

Hypertension is closely related to CNS diseases. In our study, the univariate analysis showed that hypertension was an independent risk factor for poor recovery in NMOSD patients (OR 2.86, 95%CI 1.14–7.16, *P* = 0.025), but there was no significant correlation in multivariate analysis, similar to the findings of previous studies (43). Previous studies showed that the homocysteine concentration in male patients was significantly higher than that in female patients (*P* < 0.001) (39). In our study, the gender ratio of NMOSD patients differed significantly between the normal and high Hcy groups (female, 93.9 vs. 67.9%, *P* < 0.001). The proportion of men in the high Hcy group was significantly higher than that in the other group, which requires further investigation. In addition, the univariate and multivariate analysis showed that the initial EDSS score, that is, the severity of the first attack, significantly affected the prognosis of the disease (OR 2.77, 95%CI 1.96–3.90, *P* < 0.001), which was consistent with previous research results, suggesting the importance of early treatment to improve the prognosis (44).

Previous studies have shown that, compared with a healthy control group, multiple sclerosis patients have higher Hcy levels, lower vitamin B12 and folic acid levels. Severe disability is significantly correlated with high Hcy and low vitamin B12 levels in multiple sclerosis patients (20, 36, 45, 46). In our study, folic acid in the high Hcy NMOSD group was significantly lower than in the normal Hcy group (median 6.98 vs. 9.74, *P* = 0.002). Univariate analysis showed that folic acid level had a moderate but non-significant effect on disease relapse (HR 0.97, 95%CI 0.93–1.01, *P* = 0.193), and vitamin B12 and folic acid level had no effect on the prognosis of NMOSD. This may be related to different living habits, eating habits, and drug intake of patients. The relationship between serum folic acid and vitamin B12 levels and the progress of NMOSD requires further exploration. Under the mediation of methionine synthetase, folic acid and vitamin B12 are necessary cofactors in the process of transforming Hcy into methionine (47). Vitamin B12 and folic acid supplementation could reduce serum Hcy levels (48). In our study, when serum Hcy level was higher than 14.525 μmol/l, the risk of relapse was high. This suggested that in clinical practice by reducing Hcy levels below the target value with administration of supplementary vitamin B12 and folic acid, clinicians may improve the prognosis of NMOSD and reduce the risk of relapse; this could also be used as a potential treatment strategy for NMOSD.

This research had some limitations. First, because this study was a single-center cohort study with few subjects, it is necessary to verify our results in a large, multi-center study. Second, only a small number of our patients were included in the cohort (161/544), which may have led to selection bias. Third, some patients were not tested for anti-AQP4 antibodies as our hospital started conducing anti-AQP4 antibody tests only after June 2013. Finally, some other confounding factors may affect the correlation of Hcy, vitamin B12, and folic acid, with NMOSD, such as vitamin D level, drug intake, living habits (smoking, drinking, excessive intake of coffee), etc. The inaccuracy of these data may have influenced results.

In conclusion, no previous study had reported investigation of the correlation between serum Hcy level and NMOSD progression. Our results show that serum Hcy levels are an independent risk factor for relapse and poor prognosis of NMOSD. Early monitoring and reducing the serum Hcy level may be of great significance for preventing disease relapse and severe disability. The mechanism underlying the effect of serum Hcy, folic acid, and vitamin B12 on disease progression requires further elucidation.

## Data Availability Statement

The original contributions presented in the study are included in the article/Supplementary Material, further inquiries can be directed to the corresponding author/s.

## Ethics Statement

The studies involving human participants were reviewed and approved by the Ethics Committee of Zhengzhou University. Written informed consent to participate in this study was provided by the participants' legal guardian/next of kin. Written informed consent was obtained from the individual(s), and minor(s)' legal guardian/next of kin, for the publication of any potentially identifiable images or data included in this article.

## Author Contributions

JZ and YL contributed to conception and design of the research and conducted regular follow-up of all cases. YZho, YZha, and HX organized the database. RD, YY, and JZ performed the statistical analysis. JZ wrote the first draft of the manuscript. ZG, JT, and YJ undertook the task of revising the manuscript critically. All authors contributed to manuscript revision, read, and approved the submitted version.

## Conflict of Interest

The authors declare that the research was conducted in the absence of any commercial or financial relationships that could be construed as a potential conflict of interest.
